# Response Surface Methodology (RSM)-Based Optimization of Ultrasound-Assisted Extraction of Sennoside A, Sennoside B, Aloe-Emodin, Emodin, and Chrysophanol from *Senna alexandrina* (Aerial Parts): HPLC-UV and Antioxidant Analysis

**DOI:** 10.3390/molecules27010298

**Published:** 2022-01-04

**Authors:** Perwez Alam, Omar M. Noman, Rashed N. Herqash, Omer M. Almarfadi, Ali Akhtar, Ali S. Alqahtani

**Affiliations:** 1 Department of Pharmacognosy, College of Pharmacy, King Saud University, P.O. Box 22452, Riyadh 11495, Saudi Arabia; onoman@ksu.edu.sa (O.M.N.); rhergash@ksu.edu.sa (R.N.H.); oalmarfadi@ksu.edu.sa (O.M.A.); 2 College of Pharmacy, King Saud University, Riyadh 11451, Saudi Arabia; aakhtar@ksu.edu.sa

**Keywords:** Box–Behnken design, *S. alexandrina*, sennoside A, sennoside B, aloe-emodin, emodin, chrysophanol, HPLC analysis, antioxidant analysis

## Abstract

In this study, ultrasound-assisted extraction conditions were optimized to maximize the yields of sennoside A, sennoside B, aloe-emodin, emodin, and chrysophanol from *S. alexandrina* (aerial parts). The three UAE factors, extraction temperature (*S*_1_), extraction time (*S*_2_), and liquid to solid ratio (*S*_3_), were optimized using response surface methodology (RSM). A Box–Behnken design was used for experimental design and phytoconstituent analysis was performed using high-performance liquid chromatography-UV. The optimal extraction conditions were found to be a 64.2 °C extraction temperature, 52.1 min extraction time, and 25.2 mL/g liquid to solid ratio. The experimental values of sennoside A, sennoside B, aloe-emodin, emodin, and chrysophanol (2.237, 12.792, 2.457, 0.261, and 1.529%, respectively) agreed with those predicted (2.152, 12.031, 2.331, 0.214, and 1.411%, respectively) by RSM models, thus demonstrating the appropriateness of the model used and the accomplishment of RSM in optimizing the extraction conditions. Excellent antioxidant properties were exhibited by *S. alexandrina* methanol extract obtained using the optimized extraction conditions with a DPPH assay (IC50 = 59.7 ± 1.93, µg/mL) and ABTS method (47.2 ± 1.40, µg/mL) compared to standard ascorbic acid.

## 1. Introduction

Leguminosae is one of the most widespread plant families in the world, containing the genus *Senna*, which includes hundreds of plant species [1] such as *Senna alexandrina*, which is commonly known as *Senna makkai* or *Cassia angustifolia*. This species is native to Saudi Arabia, Egypt, and Yemen [2]. It is widely used in folk medicine for several purposes including as a purgative, antipyretic, laxative, and diuretic [3]. Several other proven and relevant biological activities such as anti-allergic, anti-inflammatory, antioxidant, antibacterial, antimicrobial, analgesic, antiparasitic, insecticidal, antitumor, hepatoprotective, and antifungal properties have been reported for several species of *Senna* [4]. The medicinal properties of *Senna* can be attributed mainly to the presence of anthraquinone glycosides, especially sennosides A and B [5], which act as pro-drugs for the laxative-acting anthrones and anthraquinones [6]. Sennosides A–D are mainly responsible for the purgative action of *Senna* [7], as intestinal bacteria metabolize sennosides to rheinanthrone, which acts as a direct purgative in the intestine [8]. Sennosides have been registered as one of the leading natural pharmaceutical preparations due to their important properties [9]. The content of sennosides A and B in raw materials determine their price and acceptability on the market [10]. Other important phytochemical constituents present in *S. alexandrina* include emodin, aloe-emodin, and chrysophanol, all of which have been reported to have strong antioxidant properties [11,12].

Extraction is an important means of isolating, identifying, and applying valuable chemical compounds from natural plants [13,14]. There are many extraction methods, including water extraction, maceration extraction, and solid-phase microextraction. Typically, these traditional extraction methods are very slow, costly, and inefficient. However, several new extraction methods have been discovered during recent years. In particular, ultrasound-assisted extraction (UAE) is considered superior for natural product extraction and has been used to extract compounds from various sources. Its main advantages are its lower cost and higher efficiency compared to traditional extraction methods [15]. UAE is based on the principle of acoustic cavitation, which is capable of damaging the cell walls of the plant matrix, thereby favoring the release of bioactive compounds [16]. This technology can be applied to obtain different phytochemicals, of which phenolic compounds are a particularly notable example. These have important applications in various fields of industry, particularly the food and pharmaceutical industries, thanks to their antimicrobial, anti-inflammatory, and anticancer properties, and primarily their antioxidant capabilities [17].

**Figure 1:**
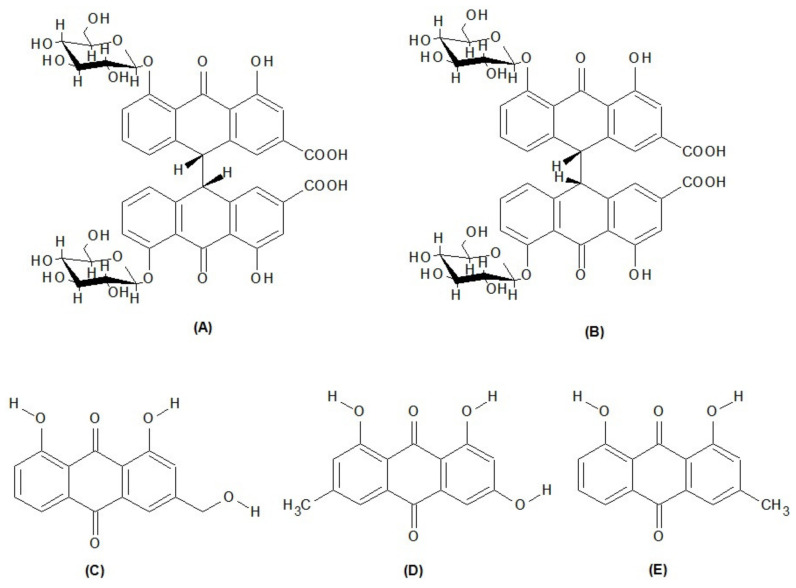
Chemical structure of phytoconstituents: (**A**) sennoside A; (**B**) sennoside B; (**C**) aloe-emodin; (**D**) emodin; (**E**) chrysophanol.

However, how to optimize extraction conditions to maximize compound yields remains challenging. In this study, we investigated the optimal extraction conditions for UAE of sennoside A, sennoside B, emodin, aloe emodin, and chrysophanol (Figure 1) through response surface methodology (RSM). RSM, which was first introduced by Box and Wilson in 1951 [18], is a collection of statistical and mathematical techniques that have been successfully used to develop, improve, and optimize processes [19,20]. RSM can be used to evaluate the effects of multiple factors and their interactions with one or more response variables [21,22]. Different RSM methods, such as the Box–Behnken design (BBD), central composite design (CCD), and three-level full factorial design (TFFD), have been widely used [23].

Several studies have previously been conducted for the extraction and quantification of active *Senna* constituents. However, the extraction optimization of important phytoconstituents sennoside A, sennoside B, aloe-emodin, emodin, and chrysophanol from *Senna* has not previously been undertaken. On this basis, we aimed to study the effects of different UAE extraction variables (extraction temperature, extraction time, and solid to liquid ratio) on the concentrations of sennoside A, sennoside B, aloe-emodin, emodin, and chrysophanol using RSM. In addition, we also aimed to develop a validated HPLC method for the concurrent analysis of sennoside A, sennoside B, aloe-emodin, emodin, and chrysophanol in optimized *S. alexandrina* samples.

## 2. Results

### 2.1. Effect of Single-Factor Tests on Total Extraction Yield of S. alexandrina (Aerial Parts)

**Figure 2:**
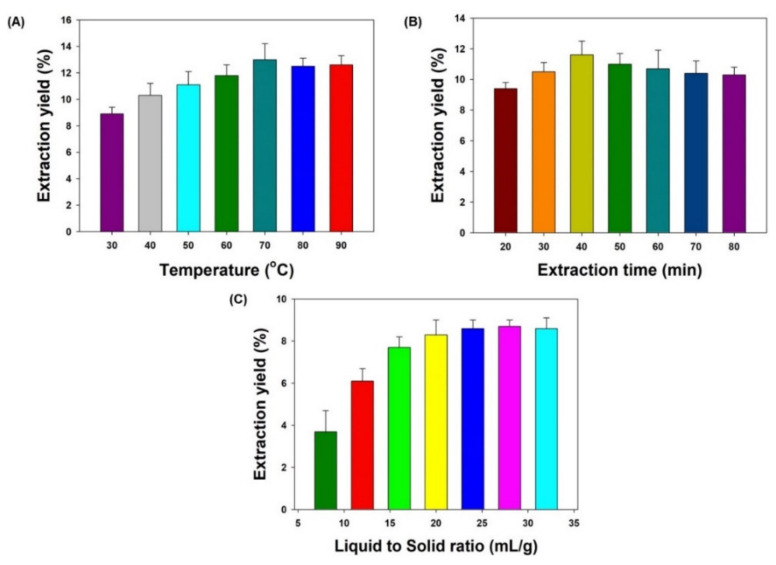
The effects of single factors on total extraction yield of *S. alexandrina* methanol extract (SAME): (**A**) extraction temperature effect; (**B**) extraction time effect; (**C**) liquid to solid ratio effect. Each value represents a mean ± SD (*n* = 5).

To set a range of UAE independent variables (extraction temperature, extraction time, and liquid to solid ratio) for optimization using the BBD method, we investigated the maximum single factor effects on the total percentage yield of *S. alexandrina*. Different ranges of extraction temperature (30–90 °C), extraction time (20–80 min), and solid to liquid ratio (8–32 mL/g) were selected to investigate the effect of individual independent factors (i.e., single factors) on total percentage yield while keeping the other two variables constant. Where these parameters were kept constant, an extraction temperature of 40 °C, extraction time of 30 min, and liquid to solid ratio of 20 mL/g were used. The results obtained showed that the total extraction yield was lowest at 30 °C extraction temperature, 20 min extraction time, and 8 mL/g liquid to solid ratio; the yields were highest at 70 °C, 40 min, and 24 mL/g, respectively. Setting the extraction temperature, extraction time, and liquid to solid ratio higher did not produce significant changes in yields (Figure 2). The increase in temperature decreases the viscosity and surface tension of the solvent, increasing its matrix penetration power and resulting in enhanced extraction. Based on these observations, the independent extraction variable ranges were set as 50–70 °C extraction temperature, 30–60 min extraction time, and 16–32 mL/g of liquid to solid ratio; extraction optimization was then applied by the BBD method.

### 2.2. BBD Method Optimization of Extraction Conditions

The ranges for the three independent extraction variables, namely, extraction temperature (*S*_1_), extraction time (*S*_2_), and liquid to solid ratio (*S*_3_), at three levels (+1, 0, −1) for extraction parameter optimization by the BBD method were selected based on observations from the single-factor experiments.

#### 2.2.1. Statistical Analysis and Model Fitting

In Table 1, the results of 17 experimental combinations of three independent extraction variables were recorded to investigate their impact on percentage yields of sennoside A (*R*_1_), sennoside B (*R*_2_), aloe-emodin (*R*_3_), emodin (*R*_4_), and chrysophanol (*R*_5_). The results were fitted into Equation (1) (i.e., a second-order polynomial equation):

(1) $$Y=\beta_{0}+\overset{n}{\underset{i=1}{\sum }}\beta_{i}P_{i}+\underset{j\;>\;1}{\overset{n-1}{\underset{i=1}{\sum }}}\overset{n}{\underset{j=2}{\sum }}\beta_{ij}\;P_{i}P_{j\;}+\overset{n}{\underset{i=1}{\sum }}\beta_{ii}P_{i\;\;}^{2}+ɛ$$

(where, Y = dependent variable (%yield); *β*_0_, *β_i_*, *β_ii_*, and *β_ij_* are the constant coefficient, linear coefficient of input factor *P_i_*, quadratic coefficient of input factor *P_i_*, and different interaction coefficients between input factor *P_i_* and *P_j_*, respectively; ɛ is the error of model) to generate the following equations with coded factors for dependent variables (*R*_1_–*R*_5_):

*R*_1_ = 2.1 + 0.1839 *S*_1_ + 0.0590 *S*_2_ + 0.0171 *S*_3_ − 0.0368 *S*_1_*S*_2_ + 0.0210 *S*_1_*S*_3_ + 0.0048 *S*_2_*S*_3_ − 0.2109 *S*_1_^2^ − 0.0681 *S*_2_^2^ − 0.1098 *S*_3_^2^.

*R*_2_ = 11.68 + 1.07 *S*_1_ + 0.4296 *S*_2_ − 0.0037 *S*_3_ − 0.1520 *S*_1_*S*_2_ + 0.1173 *S*_1_*S*_3_ + 0.2493 *S*_2_*S*_3_ − 1.06 *S*_1_^2^ − 0.4894 *S*_2_^2^ − 0.7301 *S*_3_^2^.

*R*_3_ = 2.27 + 0.2085 *S*_1_ + 0.0596 *S*_2_ + 0.0184 *S*_3_ − 0.0295 *S*_1_*S*_2_ + 0.0225 *S*_1_*S*_3_ + 0.0058 *S*_2_*S*_3_ − 0.2282 *S*_1_^2^ − 0.0735 *S*_2_^2^ − 0.1205 *S*_3_^2^.

*R*_4_ = 0.2084 + 0.0183 *S*_1_ + 0.0058 *S*_2_ + 0.0020 *S*_3_ − 0.0023 *S*_1_*S*_2_ + 0.0023 *S*_1_*S*_3_ − 0.0002 *S*_2_*S*_3_ − 0.0193 *S*_1_^2^ − 0.0073 *S*_2_^2^ − 0.0118 *S*_3_^2^.

*R*_5_ = 1.37 + 0.1220 *S*_1_ + 0.0360 *S*_2_ + 0.0150 *S*_3_ − 0.0140 *S*_1_*S*_2_ + 0.0080 *S*_1_*S*_3_ + 0.0015 *S*_2_*S*_3_ − 0.1222 *S*_1_^2^ − 0.0362 *S*_2_^2^ − 0.0583 *S*_3_^2^.

For the BBD-based experimental design, a quadratic model with an *R*^2^ value of 0.9817, 0.9850, 0.9858, 0.9895, and 0.9199 was found to be the best-fit model for the analysis of *R*_1_, *R*_2_, *R*_3_, *R*_4_, and *R*_5_, respectively. In Table 2, the regression analysis and response regression equation data for the suggested model are listed for *R*_1_, *R*_2_, *R*_3_, *R*_4_, and *R*_5_.

The adjusted *R*^2^/predicted *R*^2^ values for *R*_1_, *R*_2,_ *R*_3_, *R*_4,_ and *R*_5_ (0.9583/0.7795, 0.9656/0.8281, 0.9675/0.8462, 0.9760/0.8857, and 0.8169/0.7273, respectively) were found to be close to 1, showing a strong correlation between adjusted and predicted values. Furthermore, the difference between adjusted and predicted *R*^2^ values was found to be less than 0.2 for every dependent variable, indicating that the models fitted well. To measure whether the precision is adequate, the signal-to-noise ratio can be used, which should be greater than 4 to fit the model. In this experiment, the signal to noise ratio for *R*_1_, *R*_2_, *R*_3_, *R*_4,_ and *R*_5_ were 18.422, 20.067, 20.368, 23.618, and 8.202, respectively; all the values were more than 4, indicating that the models were fitted correctly and suggesting that the proposed model can be used to navigate the design space. In Table 3, the ANOVA (analysis of variance) results for the quadratic models of *R*_1_, *R*_2,_ *R*_3_, *R*_4_*,* and *R*_5_ are listed.

The model F-value for *R*_1_ was found to be 41.81, implying that the model was significant and that there was only a 0.01% chance that an F-value this large could occur due to noise. Similarly, for *R*_2,_ *R*_3_, *R*_4_, and *R*_5_ the F-values were found to be 50.97, 53.94, 73.19, and 8.93, respectively, implying that each quadratic model was significant and there was only a 0.01, 0.01, 0.01, and 0.43% chance, respectively, that these F-values could occur due to noise. The *p*-values for the proposed quadratic model of all the dependent variables were found to be very low (<0.05), suggesting that the models developed for analysis of all the variables were significant. The lack of fit F-values of 3.57, 2.89, 2.38, 2.42, and 0.20 for *R*_1_, *R*_2,_ *R*_3_, *R*_4,_ and *R*_5_ imply that the lack of fit is not significant relative to the pure error; thus, it is appropriate to fit the model and predict the responses. There is a 12.52, 16.61, 21.04, 20.63, and 89.48% chance that a lack of fit F-value this large could occur due to noise for variables *R*_1_, *R*_2_, *R*_3_, *R*_4,_ and *R*_5_, respectively.

#### 2.2.2. Effect of Independent Variables (*S*_1_, *S*_2_, and *S*_3_) of Ultrasonic Extraction on the Yield of *R*_1_, *R*_2,_ *R*_3_, *R*_4_, and *R*_5_

The influences of individual independent variables *S*_1_, *S*_2_, and *S*_3_, as well as their various interactions with *R*_1_, *R*_2_, *R*_3_, *R*_4_, and *R*_5_, are listed in Table 4. The linear effects of two variables (*S*_1_, *S*_2_) and quadratic effects of all three variables (*S*_12_, *S*_22_, and *S*_32_) were found to be significant (*p* < 0.05), and affect the yields of *R*_1_, *R*_2_, *R*_3_, *R*_4_, and *R*_5_. The linear effect of the liquid to solid ratio (*S*_3_) as well as the interaction effects of all three variables (*S*_1_*S*_2_, *S*_1_*S*_3_, and *S*_2_*S*_3_) exhibited no significant effect (*p* > 0.05) on the yield of any of the dependent variables (*R*_1_, *R*_2_, *R*_3_, *R*_4_, and *R*_5_). The high F-values of the linear and quadratic effects of *S*_1_ indicated that, among all the dependent variables, *S*_1_ has the most impact on the extraction dependent variables. Thus, the extraction will increase most with increasing extraction temperature but at high temperatures, the extraction will decrease.

The *R*^2^/percentage coefficient of variation values for all dependent variables (*R*_1_, *R*_2_, *R*_3_, *R*_4_, and *R*_5_) were 0.9817/2.06, 0.9850/1.96, 0.9858/1.85, 0.9895/1.53, and 0.9199/4.14, respectively, demonstrating good precision and reliability of the experimental values [24].

**Figure 3:**
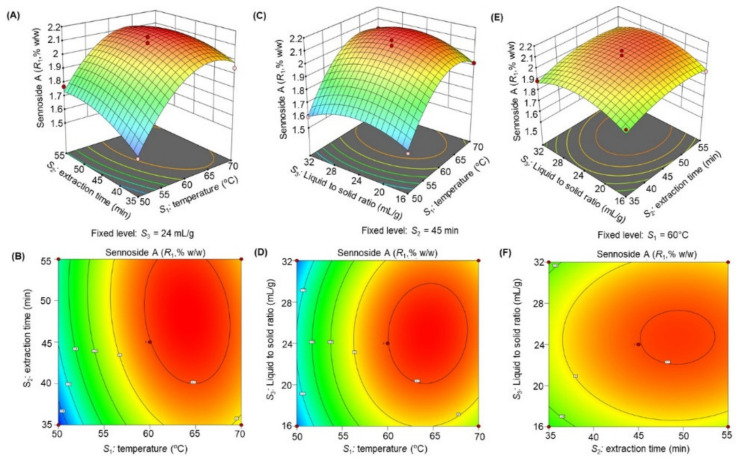
(**A**–**F**): Response surface 3D plots and 2D contour plots showing the interaction effects of temperature (*S*_1_), extraction time (*S*_2_), and liquid to solid ratio (*S*_3_) on the yields of sennoside A (*R*_1_).

**Figure 4:**
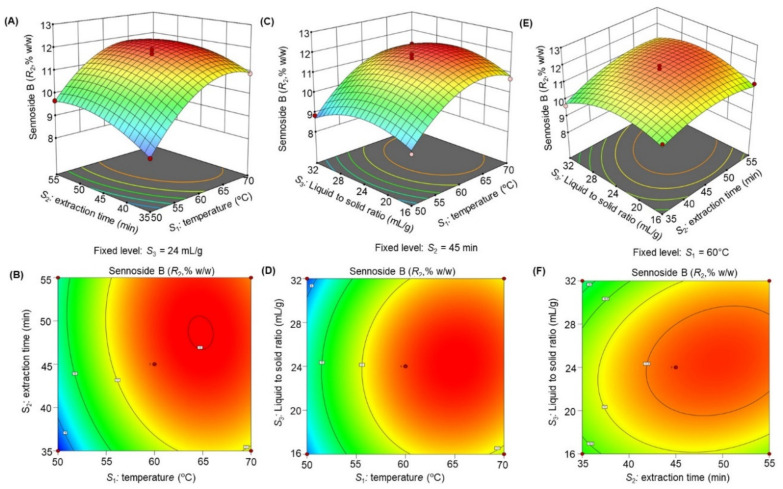
(**A**–**F**): Response surface 3D plots and 2D contour plots showing the interaction effects of temperature (*S*_1_), extraction time (*S*_2_), and liquid to solid ratio (*S*_3_) on the yields of sennoside B (*R*_2_).

**Figure 5:**
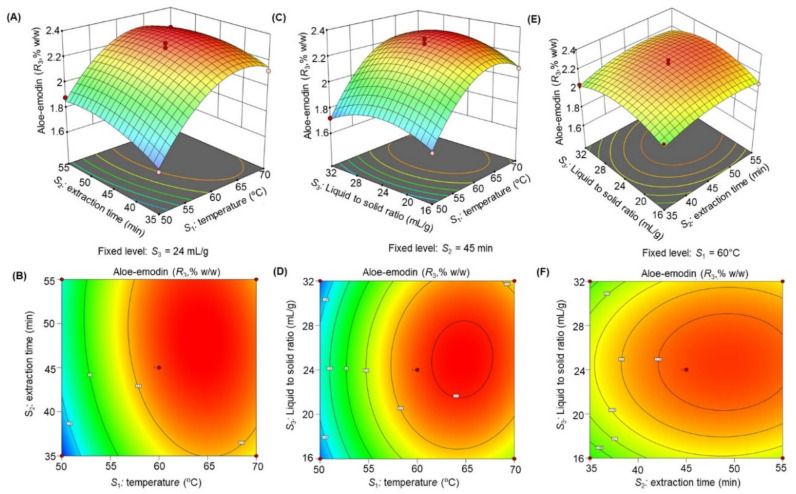
(**A**–**F**): Response surface 3D plots and 2D contour plots showing the interaction effects of temperature (*S*_1_), extraction time (*S*_2_), and liquid to solid ratio (*S*_3_) on the yields of aloe-emodin (*R*_3_).

**Figure 6:**
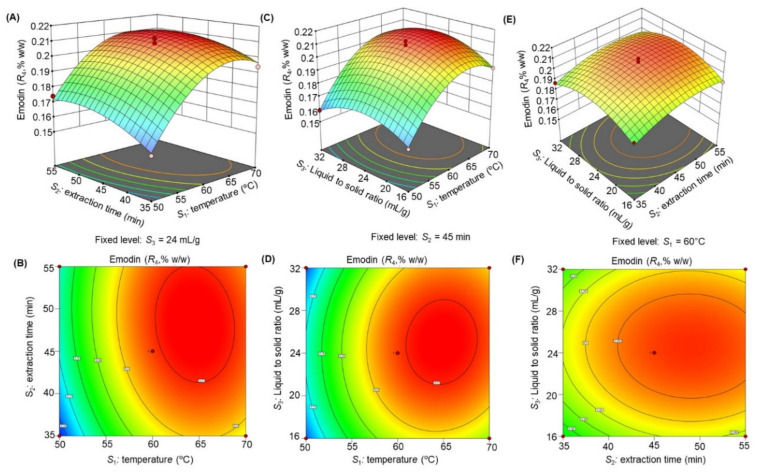
(**A**–**F**): Response surface 3D plots and 2D contour plots showing the interaction effects of temperature (*S*_1_), extraction time (*S*_2_), and liquid to solid ratio (*S*_3_) on the yields of emodin (*R*_4_).

**Figure 7:**
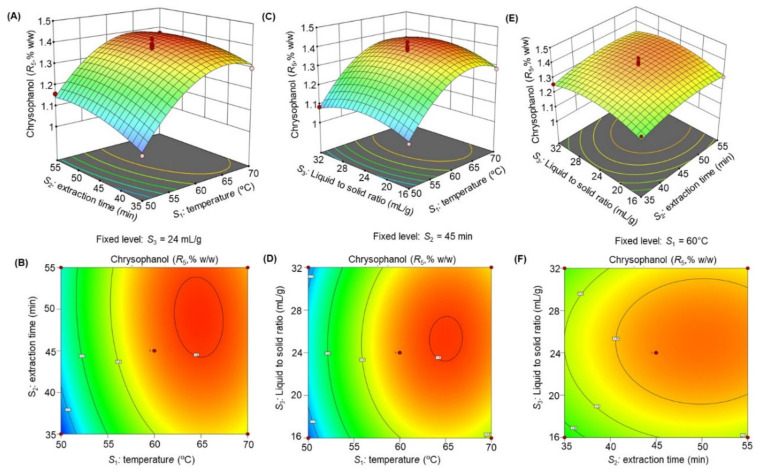
(**A**–**F**): Response surface 3D plots and 2D contour plots showing the interaction effects of temperature (*S*_1_), extraction time (*S*_2_), and liquid to solid ratio (*S*_3_) on the yields of chrysophanol (*R*_5_).

The response surface 3D plots and 2D contour plots (Figure 3, Figure 4, Figure 5, Figure 6 and Figure 7) were designed to show the interaction effects of the independent variables (*S*_1_*, S*_2_*,* and *S*_3_) on the yields of the dependent variables (*R*_1_, *R*_2_, *R*_3_, *R*_4_, and *R*_5_). Each panel demonstrates the effect of two factors on the extraction yields while the third factor was fixed at a base level (60 °C for *S*_1_, 45 min for *S*_2_*,* and 24 mL/g for *S*_3_). The effects of *S*_1_ and *S*_2_ (sub-figures A, B in Figure 3, Figure 4, Figure 5, Figure 6 and Figure 7), *S*_1_ and *S*_3_ (sub-figures C, D in Figure 3, Figure 4, Figure 5, Figure 6 and Figure 7), and *S*_2_ and *S*_3_ (sub-figures E, F in Figure 3, Figure 4, Figure 5, Figure 6 and Figure 7) on *R*_1_, *R*_2_, *R*_3_, *R*_4_, and *R*_5_ were recorded. Sub-figures A, B in Figure 3, Figure 4, Figure 5, Figure 6 and Figure 7 showed that the extraction yields of the dependent variables were highest at *S*_1_ of 63.8 °C and *S*_2_ of 51.6 min. When *S*_1_ exceeded 63.8 °C, the yield was found to decrease. The extraction yields of *R*_1_–*R*_5_ increased when *S*_1_ was set at 63.5 °C and *S*_3_ at 25.6 mL/g, as shown in sub-figures C, D in Figure 3, Figure 4, Figure 5, Figure 6 and Figure 7. Sub-figures E, F in Figure 3, Figure 4, Figure 5, Figure 6 and Figure 7 demonstrate the effect of *S*_2_ and *S*_3_ interaction on the dependent variables*;* no significant changes were recorded with an increase in *S*_3_, however, a significant increase was observed with increasing *S*_2_. Based on these observations, we concluded that the maximal *R*_1_, *R*_2_, *R*_3_, *R*_4_, and *R*_5_ could be extracted from the aerial parts of *S. alexandrina* using UAE at a temperature of 63.8 °C, extraction time of 51.6 min, and a liquid to solid ratio of 25.6 mL/g.

#### 2.2.3. BBD Method Validation

The validation of the BBD method used for the analysis was performed by comparing the experimental values and predicted values of the responses (*R*_1_, *R*_2_, *R*_3_, *R*_4_, and *R*_5_). Establishing the suitability of the generated polynomial equation and BBD application was performed using the percentage prediction error, where small percentage prediction error values demonstrate the validity of the generated polynomial equation and application of the BBD model. The linear correlation between actual and predicted values of *R*_1_, *R*_2_, *R*_3_, *R*_4_, and *R*_5_ demonstrated high *R*^2^ values of 0.9923, 0.9949, 0.9918, 0.9972, and 0.9941, respectively, indicating excellent goodness of fit (*p* < 0.001). 

### 2.3. HPLC Analysis of BBD Optimized SAME

**Figure 8:**
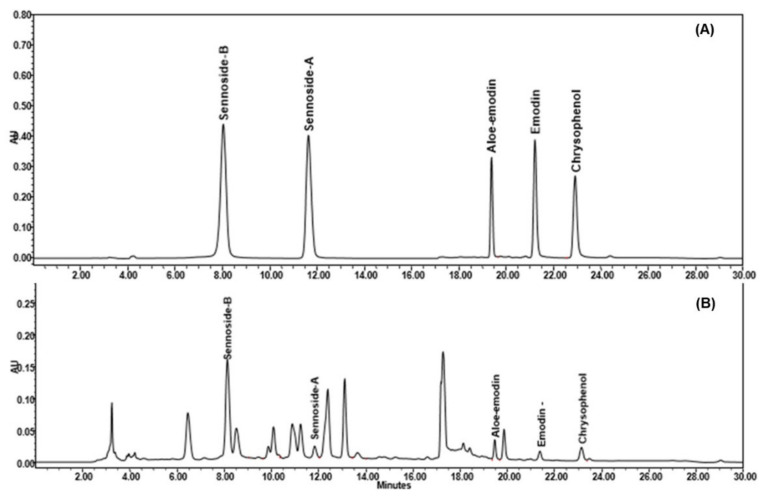
Chromatogram of sennoside A, sennoside B, aloe-emodin, emodin, and chrysophenol estimation in SAME by HPLC-UV method [Conditions: Pinnacle C18 column (4.6 × 250 mm, 5 µm); mobile phase, acetonitrile: 0.5% formic acid in ultra-pure water (gradient system); flow rate: 1 mL/min; UV-detection at λmax = 272 nm at temperature (25 ± 1 °C)]. (**A**) Representative chromatogram of sennoside B (Rt = 8.078), sennoside A (Rt = 11.79), aloe-emodin (Rt = 19.43), emodin (Rt = 21.25), and chrysophenol (Rt = 22.98). (**B**) Representative chromatogram of SAME containing sennoside B, sennoside A, aloe-emodin, emodin, and chrysophenol.

A pinnacle DB Aqueous C18 reversed-phase packing column (Bellefonte, Pennsylvania, United States; 4.6 × 250 mm, 5 μm) was used to separate sennoside A (*R*_1_), sennoside B (*R*_2_), aloe-emodin (*R*_3_), emodin (*R*_4_), and chrysophanol (*R*_5_), along with the various phytoconstituents available in *S. alexandrina* methanol extract (SAME) using 0.5% formic acid in ultra-pure water and acetonitrile (a gradient elution) as a mobile phase. Figure 8A illustrates the separation of *R*_1_–*R*_5_ at 272 nm for quantitative analysis. A robust baseline separation was achieved in 26 min. Figure 8B demonstrates a representative chromatogram of *S. alexandrina* (aerial parts) extracted with methanol by the optimized ultrasonication method. In this experiment, we used the gradient system for elution of the standards and SAME as this approach increases the elution strength, sensitivity, and efficiency of the HPLC column, improves separation quality and detection limit, and decreases the analysis time and column degradation due to strongly retained analytes. Under these conditions, the retention times of sennoside B, sennoside A, aloe-emodin, emodin, and chrysophanol were found to be 8.078, 11.79, 19.43, 21.25, and 22.98 min, respectively.

The developed HPLC method yielded high linearity for sennoside A, sennoside B, aloe-emodin, emodin, and chrysophenol (r^2^ = 0.999, each) in the 0.5–20 µg/mL linearity range, and low limit of detection (LOD)/limit of quantification (LOQ) (µg/mL) values for sennoside A (0.09/0.29), sennoside B (0.009/0.03), aloe-emodin (0.07/0.23), emodin (0.09/0.273), and chrysophenol (0.019/0.06). The intraday/interday precision (%RSD, relative standard deviation) was recorded at different concentration levels (5, 10, and 15, µg/mL) and found to be: 4.84–5.81%/3.83–4.74% for sennoside A; 3.84–5.41%/3.03–4.81% for sennoside B; 3.22–4.88%/2.42–4.55% for aloe-emodin; 2.80–4.07%/2.20–3.81% for emodin; and 4.21–4.81%/3.62–4.61% for chrysophenol. Such low precision values for the standards indicate that the method is repeatable.

### 2.4. Verification of Optimized Microwave-Assisted Extraction Conditions

The selected factors (*S*_1_–*S*_3_) demonstrated diverse effects on the yield of dependent variables (*R*_1_–*R*_5_) (Table 5). The predicted optimal levels for *R*_1_–*R*_5_ extraction were found to be *S*_1_ of 63.8 °C, *S*_2_ of 51.6 min, and *S*_3_ of 25.6 mL/g, which were predicted to yield (% *w*/*w*) *R*_1_ (2.152), *R*_2_ (12.031), *R*_3_ (2.331), *R*_4_ (0.214), and *R*_5_ (1.411). The predicted optimal level for each extraction factor was further modified to *S*_1_ of 64.2 °C, *S*_2_ of 52.1 min, and *S*_3_ of 25.2 mL/g to obtain the maximum yields of *R*_1_–*R*_5_. The modified level of all extraction factors was used (*n* = 3) for the extraction of aerial parts of *S. alexandrina* using methanol (SAME) and the analysis of the obtained SAME was carried out using the developed HPLC-UV method to quantify *R*_1_–*R*_5_. By using the modified extraction condition, the amounts (% *w*/*w*) of *R*_1_, *R*_2_, *R*_3_, *R*_4_, and *R*_5_ in SAME were found to be 2.237 ± 0.051%, 12.792 ± 0.475%, 2.457 ± 0.053%, 0.261 ± 0.0052%, and 1.529 ± 0.041%, respectively. The low residual percentages of 1.04, 1.06, 1.05, 1.22, and 1.08% for *R*_1_, *R*_2_, *R*_3_, *R*_4_, and *R*_5_, respectively, indicated that the model was reliable.

### 2.5. 2,2-Diphenyl-1-Picrylhydrazyl (DPPH) and 2,2′-Azino-Bis (3-Ethylbenzothiazoline-6-Sulfonic Acid (ABTS) Free Radical Scavenging Activities of SAME Samples

The DPPH radical scavenging activity for the 17 (S1–S17) SAME samples, along with the final BBD-optimized ultrasonic-extracted SAME, were evaluated, and the IC50 (µg/mL) values are shown in Table 6. Among these 17 SAME samples (S1–S17), many exhibited good antioxidant properties. Out of these 17 samples, S5 showed the maximum free radical scavenging property (IC50 = 68.4 ± 2.18 µg/mL) compared with standard ascorbic acid (IC50 = 5.9 ± 0.23 µg/mL). The SAME obtained after using the BBD-optimized ultrasonic extraction parameters exhibited strong antioxidant properties, having IC50 = 59.7 ± 1.93 µg/mL. Both the S5 and BBD-optimized SAME were found to contain a substantial amount of anthraquinone glycosides (sennoside A, sennoside B, aloe-emodin, emodin, and chrysophanol), as evaluated by using the HPLC-UV method, which may explain the samples’ antioxidant properties.

The results of ABTS radical scavenging activity of all SAME samples (S1–S17) as well as BBD-optimized ultrasonic-extracted samples given in Table 6 clearly indicate that the extracts (S5 and BBD-optimized SAME) containing larger amounts of sennoside A, sennoside B, aloe-emodin, emodin, and chrysophanol (evaluated by HPLC-UV method) had greater free radical-scavenging properties (IC50 = 58.9 ± 2.33 and 47.2 ± 1.40 µg/mL, respectively). The optimization of extraction parameters leads to an increase in the extraction of all the standards (evaluated by HPLC analysis), which supported the increase in the free radical-scavenging property of the extract. Al-Zain et al. [25] found DPPH and ABTS antioxidant properties of methanol extract of *S. alexandrina* root with IC50 values of 235.9 and 321.5 µg/mL, respectively. Ahmed et al. [2] reported free radical-scavenging properties of methanol extract of *C. angustifolia* leaves with IC50 = 2.49 ± 0.01 µg/mL.

## 3. Discussion

In this experiment, we investigated the effects of various ultrasonic extraction parameters (extraction temperature, extraction time, and liquid to solid ratio) on sennoside A, sennoside B, aloe-emodin, emodin, and chrysophanol extraction from aerial parts of *S. alexandrina* using the BBD of RSM. The ultrasonication technique was previously reported to be the best method for sennoside A and sennoside B extraction from *C. angustifolia* leaves [26] as well as for the extraction of aloe-emodin, emodin, and chrysophanol from *Rhamnus alpinus* bark [27]. This was the basis of exploiting the ultrasonication extraction method and optimizing the extraction parameters by the BBD approach to obtain maximum yields from aerial parts of *S. alexandrina*. The BBD of RSM plays an important role in determining the optimal values of independent variables under which the maximum response of dependent variables can be achieved [28]. The response surface and contour plot approaches were used to visualize the correlation between responses and different levels of independent variables and interaction types between two independent variables [29]. In this experiment, at a fixed liquid to solid ratio and extraction time, the yields of sennoside A, sennoside B, aloe-emodin, emodin, and chrysophanol increased with increasing extraction temperatures. Similar results were reported by Zhao et al. [30] and Ruan et al. [31], which may indicate that the solubility of these substances increases with increased temperature and, thus, the total yields also increase. Furthermore, the sennoside A, sennoside B, aloe-emodin, emodin, and chrysophanol yields increased with increasing extraction time to a maximum, followed by a decline with further increases in extraction time. The decrease in extraction yields may be due to degradation of the phytoconstituents upon prolonged ultrasound action [32]. Mason et al. [33] reported that ultrasound may induce the formation of acoustic cavities, which prompted cracking of the plant cells. The plant cells cracked completely with increasing extraction time due to acoustic cavitation, thus increasing the extraction yields of the desired compounds. However, insoluble constituents, along with cytosol, become suspended in extraction solvent when the plant cells rupture, resulting in a reduction of the solvent permeability [34]. Additionally, the desired constituents become re-adsorbed on the ruptured plant particles because of their comparatively more specific surface areas, thus decreasing the yields [35]. A mathematical optimization was performed by using the BBD of RSM to decide the optimum level of different extraction variables to obtain maximum yields of the desired compounds. An extraction temperature of 63.8°C, 51.6 min extraction time, and a 25.6 mL/g liquid to solid ratio were determined as the optimal extraction conditions for the ultrasonication technique. The maximum response values (% *w*/*w*) were predicted as 2.152, 12.031, 2.331, 0.214, and 1.411% of sennoside A, sennoside B, aloe-emodin, emodin, and chrysophanol, respectively, under these operating conditions. The experiment was performed again with a modified extraction condition (extraction temperature of 64.2 °C, extraction time of 52.1 min, and 25.2 mL/g liquid to solid ratio) to obtain the maximum yield of the desired compounds. The extraction procedure with modified conditions was performed in triplicate, and the results of experimental values were compared with the predicted values. Mean values (% *w*/*w*) of 2.237% ± 0.051, 12.792% ± 0.475, 2.457% ± 0.053, 0.261% ± 0.0052, and 1.5297% ± 0.041, respectively, for sennoside A, sennoside B, aloe-emodin, emodin, and chrysophanol were obtained using the modified extraction procedure, which were very close to the predicted values, demonstrating the validity of the RSM model and also demonstrating that the suitability of the model for this extraction procedure.

Several phenolic substances, of both natural and synthetic origin, have been shown to act as antioxidant and radical scavengers, In particular, the flavonoids, which constitute an ubiquitous class as metabolites in higher plants, have been reported to contain numerous active substances [36,37]. In contrast, the anthraquinones and anthrones appear to be nearly unknown in this respect. However, it has been reported that substances of this class have interesting effects as antioxidants; some anthrones may have prooxidant activity due to their ability to generate superoxide anions [12]. It was found that anthraquinones such as chrysophanol and rhein accelerated the formation of hydroxyl radicals and among these compounds, emodin exhibited the strongest scavenging effect on hydroxyl radicals [38]. Emodin has been known to be pharmacologically potent and this characteristic was reported to be associated with its scavenging of hydroxyl radicals [39]. In contrast, the prooxidant ability of chrysophenol may derive from its greater formation of hydroxyl radicals [12]. In this experiment excellent antioxidant properties were exhibited by SAME obtained using the optimized extraction conditions with DPPH assay (IC50 = 59.7 ± 1.93, µg/mL) and ABTS methods (IC50 = 47.2 ± 1.40, µg/mL) compared to standard ascorbic acid. This supported the HPLC-UV analysis of antioxidant marker like emodin, chrysophenol in good quantity in SAME. There may be other compounds present in SAME along with these compounds that might support the antioxidant action of these markers synergistically.

## 4. Materials and Methods

### 4.1. Plant Material

The aerial parts of *S. alexandrina* (voucher specimen no. 16245) were collected in 2014 from Badr, Kingdom of Saudi Arabia by Dr. Md. Yusuf (field taxonomist, Pharmacognosy Department), and the specimen was deposited in the herbarium of Pharmacognosy Department, College of Pharmacy, King Saud University, Saudi Arabia. The collected aerial parts were cut into small pieces, washed with plain water, dried in the plant drying room in the Pharmacognosy Department, and stored in a tightly packed glass jar. Before extraction, the aerial parts were coarsely powdered using a grinder.

### 4.2. Apparatus and Reagents

The standard compounds sennoside A (≥96.0%), sennoside B (≥90.0%), aloe-emodin (≥95%), emodin (≥97.0%), and chrysophanol (≥98.0%) were purchased from Sigma-Aldrich (St. Louis, MO, USA). The HPLC-grade methanol and acetonitrile were procured from WINLAB (Market Harborough, UK). A Millipore Milli-Q^®^ (Bedford, MA, USA) assembly was used to obtain highly pure water. A Millipore-Millex-HV^®^ filter unit with a membrane filter (0.45 µm pore size) was used for the filtration of solvents, and a syringe filter of 0.22 µm was used for sample preparation. For quantitative analysis, an Alliance 2695 separation module equipped with a 2487 dual wavelength absorbance detector (Waters Instruments, Inc., Milford, MA, USA) was used. DPPH and ABTS reagents for antioxidant assays were procured from Sigma-Aldrich (St. Louis, MO, USA).

### 4.3. Ultrasound-Assisted Extraction of S. alexandrina Aerial Parts

The extraction of *S. alexandrina* powdered aerial parts (1 g) was executed in a 50 mL conical flask by UAE using a Sonics vibra cell (Model VCX-750; Sonics, Newtown, CT, USA) with methanol as solvent. Post-extraction, the SAME was cooled and filtered, and the residue was washed thrice with methanol to obtain the final SAME volume, which was then filtered using a syringe filter (0.45 µm, Phenomenex, Torrance, CA, USA). The final filtered extract was dried using a rotavapor (R-300, Buchi, Flawil, Switzerland) to obtain the final percentage yield.

### 4.4. BBD Experimental Design

#### 4.4.1. Single Factor Experimental Design

The extraction variables (extraction temperature, time, and liquid to solid ratio) were selected based on observation of single-factor effects on the total extraction yield, which was used to optimize all extraction variables using the BBD method to obtain maximum sennoside A, sennoside B, aloe-emodin, emodin, and chrysophanol content from SAME. The impact of single factors on total extraction yield was evaluated by varying the extraction temperatures (30–90 °C), extraction times (20–80 min), and liquid to solid ratios (8–32 mL/g). When one factor was being used as a variable, the other two factors remained fixed (extraction temperature of 40 °C, extraction time of 30 min, and liquid to solid ratio of 20 mL/g).

#### 4.4.2. Optimization of Extraction Variables Using BBD Method and Method Validity Testing

A 3-factorial (3^3^) Box–Behnken design (version 13, Design-Expert Software, Stat-Ease Inc., Minneapolis, USA) was used for optimization of the independent variables extraction temperature (*S*_1_), extraction time (*S*_2_), and liquid to solid ratio (*S*_3_), at low (−1), medium (0), and high levels (+1) (Table 7). A total of 17 experimental runs, comprising five central points, were generated and fitted to a second-order polynomial equation to obtain the total yields of sennoside A (*R*_1_), sennoside B (*R*_2_), aloe-emodin (*R*_3_), emodin (*R*_4_), and chrysophanol (*R*_5_). Two-dimensional contour plots and three-dimensional response surface plots were made to deduce the independent variables’ effects on *R*_1_, *R*_2_, *R*_3_, *R*_4_, and *R*_5_ yields, and the “biggest-is-best” principle was used for each variable to obtain the optimum outcome, with *p*-values ≤ 0.05 considered to be significant. A final confirmation experiment (*n* = 3) was performed using optimized independent extraction variables, and the experimental yields were compared with predicted values for model validation.

### 4.5. High-Performance Liquid Chromatography (HPLC) Analysis of BBD-run SAME Samples

For quantitative analysis of *R*_1_, *R*_2_, *R*_3_, *R*_4_, and *R*_5_ in all 17 BBD-run SAMEs, as well as the final extract obtained using the optimized extraction conditions, an Alliance 2695 Separations module equipped with a 2487 dual wavelength absorbance detector (Waters Instruments, Inc., Milford, MA, USA), was used. The chromatographic analysis was performed using the following equipment: built-in quaternary pump, Pinnacle C18 column (5 μm, 250 × 4.6 mm), four-channel degasser, and autosampler with programmable temperature (25 °C). Different proportions of 0.5% formic acid in ultra-pure water (A) and acetonitrile (B) were used as a mobile phase with a flow rate adjusted to 1 mL/min. The gradient program was optimized as: 0–15 min (15–30% B), 15–18 min (30–34% B), 18–20 min (34–40% B), 20–21 min (40–15% B), and 21–25 min (15% B). Samples (1 mg/mL) were injected into the system at a volume of 10 µL; the output signal was detected at λmax = 272 nm and processed using EMPOWER software version 2.

### 4.6. Antioxidant Assay of BBD-Run SAME Samples

#### 4.6.1. DPPH Radical-Scavenging Assay

The antioxidative properties of the 17 BBD-run SAMEs, as well as the final extract obtained using the optimized extraction conditions, were evaluated using DPPH, according to the earlier report by Alqahtani et al. [40], with minimal modifications. Different concentrations of the extracts (10, 50, 100, 500, and 1000 μg/mL) were prepared. Subsequently, 500 μL of each concentration was added to 125 μL of DPPH and 375 μL of methanol and then incubated for 30 min at room temperature. The antioxidant activity was measured spectrophotometrically (UV mini-1240, Shimadzu, Japan) in absorbance mode at λmax = 517 nm using ascorbic acid as the positive control. The following equation was used to calculate the radical-scavenging activity:

% of radical-scavenging activity = (Abs control − Abs sample/Abs control) × 100

#### 4.6.2. ABTS Radical Cation Scavenging Activity

The antioxidative assay of the 17 BBD-run SAMEs, as well as the final extract obtained by using the optimized extraction conditions, was evaluated by applying the ABTS method according to the previous report of Li et al. [41], with small modifications. Aqueous solutions of ABTS (7 mmol/L) and potassium persulfate (2.45 mmol/L) were prepared and, after 12 h in the dark, the two solutions were mixed and incubated for 0.5 h, kept in a refrigerator for 24 h, and then diluted in ethanol. To generate a calibration curve, a reaction was initiated by pipetting different prepared concentrations of each extract (10, 50, 100, 500, and 1000 μg/mL) to ABTS solutions of 50 µg/mL (1:1) and recording the absorbance at λmax = 734 nm using ethanol (95%), ABTS (50 µg/mL), and ascorbic acid as a blank, control, and standard, respectively. Three replicates for the standard and each extract were used for the analysis. The percentage of antioxidant property for each extract was calculated using the following formula [42]:

% of radical scavenging activity = (Abs control − Abs sample/Abs control) × 100

### 4.7. Statistical Analysis

All experiments were carried out with three independent replicates and the values are presented as mean ± standard error of the mean (SEM). Data were statistically analyzed using the Student’s *t*-test for comparison between the means, applying a significance level of *p* < 0.05.

## 5. Conclusions

In this study, RSM with a BBD was used to study ultrasound-assisted extraction of sennoside A, sennoside B, aloe-emodin, emodin, and chrysophanol from the aerial parts of *S. alexandrina*. The experimental results demonstrated that all three extraction variables (extraction temperature, extraction time, and liquid to solid ratio), had an impact on the extraction of the target compounds. The optimal/modified extraction conditions were found to be: extraction temperature 63.8/64.2 °C, extraction time 51.6/52.1 min, and liquid to solid ratio 25.6/25.2 mL/g. Under these conditions, the experimental yields (% *w*/*w*) of sennoside A, sennoside B, aloe-emodin, emodin, and chrysophanol were found to be 2.237, 12.792, 2.457, 0.261, and 1.529%, respectively, which agreed closely with predicted yields (2.152, 12.031, 2.331, 0.214, and 1.411%, respectively). The experimental values were consistent with values predicted by RSM models, thus demonstrating the fitness of the model employed and the accomplishment of RSM in optimizing the extraction conditions. An increase in the antioxidant property of the BBD optimized SAME against DPPH and ABTS radicals were supported by the increase in the content of antioxidant markers like emodin and chrysophenol (HPLC-UV anlysis). There may be some other antioxidant compounds present in BBD optimized SAME alongwith these markers which might synergistacally exhibited the excellent antioxidant propery of SAME. In the future, the proposed optimized ultrasonication extraction method in this work can be employed to extract sennoside A, sennoside B, aloe-emodin, emodin, and chrysophanol efficiently from marketed herbal supplements containing different *Senna* species.

## Figures and Tables

**Table 1:** Experimental parameters of Box–Behnken design and analysis of sennoside A (*R*_1_), sennoside B (*R*_2_), aloe-emodin (*R*_3_), emodin (*R*_4_), and chrysophanol (*R*_5_) by HPLC-UV (*n* = 3).

Run	Coded Variables			Sennoside A Yield (*R*_1_)	Sennoside B Yield (*R*_2_)	Aloe-Emodin Yield (*R*_3_)	Emodin Yield (*R*_4_)	Chrysophanol Yield (*R*_5_)
	(*S*_1_) (°C)	(*S*_2_) (min)	(*S*_3_) (mL/g)	(% *w*/*w*)	(% *w*/*w*)	(% *w*/*w*)	(% *w*/*w*)	(% *w*/*w*)
1	0	1	1	1.963 ± 0.088	10.967 ± 0.559	2.125 ± 0.056	0.194 ± 0.004	1.306 ± 0.035
2	−1	1	0	1.77 ± 0.074	9.667 ± 0.454	1.881 ± 0.064	0.174 ± 0.003	1.158 ± 0.042
3	−1	−1	0	1.524 ± 0.057	8.481 ± 0.449	1.651 ± 0.041	0.154 ± 0.005	1.028 ± 0.031
4	−1	0	−1	1.575 ± 0.067	8.763 ± 0.534	1.705 ± 0.049	0.158 ± 0.005	1.052 ± 0.048
5	0	0	0	2.143 ± 0.091	11.921 ± 0.638	2.32 ± 0.073	0.212 ± 0.008	1.424 ± 0.059
6	0	1	−1	1.941 ± 0.054	10.796 ± 0.619	2.101 ± 0.049	0.192 ± 0.007	1.291 ± 0.067
7	1	−1	0	1.942 ± 0.047	10.899 ± 0.585	2.121 ± 0.061	0.194 ± 0.006	1.297 ± 0.041
8	0	0	0	2.101 ± 0.094	11.742 ± 0.668	2.285 ± 0.079	0.209 ± 0.009	1.401 ± 0.046
9	0	0	0	2.081 ± 0.083	11.576 ± 0.714	2.252 ± 0.071	0.206 ± 0.011	1.384 ± 0.043
10	0	−1	1	1.89 ± 0.043	9.632 ± 0.461	2.046 ± 0.051	0.187 ± 0.006	1.261 ± 0.039
11	1	0	−1	1.924 ± 0.069	10.705 ± 0.574	2.083 ± 0.055	0.191 ± 0.004	1.283 ± 0.042
12	0	−1	−1	1.887 ± 0.077	10.458 ± 0.515	2.045 ± 0.064	0.184 ± 0.003	1.252 ± 0.039
13	1	0	1	2.022 ± 0.084	11.252 ± 0.707	2.189 ± 0.061	0.201 ± 0.009	1.347 ± 0.061
14	0	0	0	2.093 ± 0.085	11.643 ± 0.688	2.265 ± 0.072	0.207 ± 0.008	1.392 ± 0.057
15	0	0	0	2.073 ± 0.097	11.532 ± 0.633	2.244 ± 0.078	0.208 ± 0.01	1.259 ± 0.051
16	1	1	0	2.041 ± 0.092	11.477 ± 0.554	2.233 ± 0.055	0.205 ± 0.009	1.371 ± 0.061
17	−1	0	1	1.589 ± 0.061	8.841 ± 0.501	1.721 ± 0.046	0.159 ± 0.008	1.084 ± 0.026

**Table 2:** Regression analysis and response regression equation results for the final proposed model.

Dependent Variables	Source	*R* ^2^	Adjusted *R*^2^	Predicted *R*^2^	SD
*R*_1_	Linear	0.5019	0.3869	0.2315	0.1515
	2FI	0.5140	0.2224	−0.3570	0.1706
	Quadratic	0.9817	0.9583	0.7795	0.0395
*R*_2_	Linear	0.5285	0.4197	0.2927	0.8562
	2FI	0.5481	0.2769	−0.1593	0.9557
	Quadratic	0.9850	0.9656	0.8281	0.2083
*R*_3_	Linear	0.5228	0.4127	0.2686	0.1631
	2FI	0.5306	0.2489	−0.2998	0.1845
	Quadratic	0.9858	0.9675	0.8462	0.0384
*R*_4_	Linear	0.5215	0.4111	0.2805	0.0145
	2FI	0.5287	0.2459	−0.2491	0.0164
	Quadratic	0.9895	0.9760	0.8857	0.0029
*R*_5_	Linear	0.5439	0.4387	0.3206	0.0920
	2FI	0.5483	0.2773	−0.1764	0.1044
	Quadratic	0.9199	0.8169	0.7273	0.0525

**Table 3:** ANOVA for the fitted quadratic polynomial model of *R*_1_, *R*_2_, *R*_3_, *R*_4_, and *R*_5_.

Dependent Variables	Source	Sum of Square	Degree of Freedom	Mean Square	F-Value	*p* Value
*R*_1_	Model	0.5881	9	0.0653	41.81	<0.0001 (significant)
	Residual	0.0109	7	0.0016	-	-
	Lack of fit	0.0080	3	0.0027	3.57	0.1252 (not significant)
	Pure error	0.0030	4	0.0007	-	-
*R*_2_	Model	19.91	9	2.21	50.97	<0.0001 (significant)
	Residual	0.3038	7	0.0434	-	-
	Lack of fit	0.2078	3	0.0693	2.89	0.1661 (not significant)
	Pure error	0.0960	4	0.0240	-	-
*R*_3_	Model	0.7145	9	0.0794	53.94	<0.0001 (significant)
	Residual	0.0103	7	0.0015	-	-
	Lack of fit	0.0066	3	0.0022	2.38	0.2104 (not significant)
	Pure error	0.0037	4	0.0009	-	-
*R*_4_	Model	0.0056	9	0.0006	73.19	<0.0001 (significant)
	Residual	0.0001	7	8.529 × 10^−6^	-	-
	Lack of fit	0.0001	3	0.0001	2.42	0.2063 (not significant)
	Pure error	0.0001	4	5.300 × 10^−6^	-	-
*R*_5_	Model	0.2220	9	0.0247	8.93	0.0043 (significant)
	Residual	0.0193	7	0.0028	-	-
	Lack of fit	0.0025	3	0.0008	0.1950	0.8948 (not significant)
	Pure error	0.0169	4	0.0042	-	-

**Table 4:** The significance of each response variable effect shown by using the F ratio and *p*-value in the nonlinear second-order model.

Dependent Variables	Independent Variables	SS ^a^	DF ^b^	MS ^c^	*F*-Value	*p*-Value ^d^
*R*_1_	**Linear Effects**					
	*S*_1_	0.2705	1	0.2705	173.06	<0.0001
	*S*_2_	0.0278	1	0.0278	17.82	0.0039
	*S*_3_	0.0023	1	0.0023	1.50	0.2601
	**Quadratic Effects**					
	*S*_1_^2^	0.1872	1	0.1872	119.77	<0.0001
	*S*_2_^2^	0.0195	1	0.0195	12.49	0.0095
	*S*_3_^2^	0.0508	1	0.0508	32.51	0.0007
	**Interaction Effects**					
	*S*_1_ *S*_2_	0.0054	1	0.0054	3.46	0.1053
	*S*_1_ *S*_3_	0.0018	1	0.0018	1.13	0.3233
	*S*_2_ *S*_3_	0.0001	1	0.0001	0.0577	0.8170
*R*_2_	**Linear Effects**					
	*S*_1_	9.20	1	9.20	212.10	<0.0001
	*S*_2_	1.48	1	1.48	34.03	0.0006
	*S*_3_	0.0001	1	0.0001	0.0026	0.9608
	**Quadratic Effects**					
	*S*_1_^2^	4.75	1	4.75	109.52	< 0.0001
	*S*_2_^2^	1.01	1	1.01	23.24	0.0019
	*S*_3_^2^	2.24	1	2.24	51.73	0.0002
	**Interaction Effects**					
	*S*_1_ *S*_2_	0.0924	1	0.0924	2.13	0.1878
	*S*_1_ *S*_3_	0.0550	1	0.0550	1.27	0.2974
	*S*_2_ *S*_3_	0.2485	1	0.2485	5.73	0.0480
*R*_3_	**Linear Effects**					
	*S*_1_	0.3478	1	0.3478	236.28	<0.0001
	*S*_2_	0.0284	1	0.0284	19.32	0.0032
	*S*_3_	0.0027	1	0.0027	1.84	0.2176
	**Quadratic Effects**					
	*S*_1_^2^	0.2193	1	0.2193	149.00	<0.0001
	*S*_2_^2^	0.0227	1	0.0227	15.44	0.0057
	*S*_3_^2^	0.0611	1	0.0611	41.52	0.0004
	**Interaction Effects**					
	*S*_1_ *S*_2_	0.0035	1	0.0035	2.37	0.1680
	*S*_1_ *S*_3_	0.0020	1	0.0020	1.38	0.2792
	*S*_2_ *S*_3_	0.0001	1	0.0001	0.0899	0.7731
*R*_4_	**Linear Effects**					
	*S*_1_	0.0027	1	0.0027	312.42	<0.0001
	*S*_2_	0.0003	1	0.0003	31.01	0.0008
	*S*_3_	0.0000	1	0.0000	3.75	0.0939
	**Quadratic Effects**					
	*S*_1_^2^	0.0016	1	0.0016	184.37	<0.0001
	*S*_2_^2^	0.0002	1	0.0002	26.49	0.0013
	*S*_3_^2^	0.0006	1	0.0006	69.03	<0.0001
	**Interaction Effects**					
	*S*_1_ *S*_2_	0.0000	1	0.0000	2.37	0.1672
	*S*_1_ *S*_3_	0.0000	1	0.0000	2.37	0.1672
	*S*_2_ *S*_3_	2.500 × 10^−7^	1	2.500 × 10^−7^	0.0293	0.8689
*R*_5_	**Linear Effects**					
	*S*_1_	0.1191	1	0.1191	43.13	0.0003
	*S*_2_	0.0104	1	0.0104	3.76	0.0938
	*S*_3_	0.0018	1	0.0018	0.6520	0.4460
	**Quadratic Effects**					
	*S*_1_^2^	0.0629	1	0.0629	22.79	0.0020
	*S*_2_^2^	0.0055	1	0.0055	2.00	0.1998
	*S*_3_^2^	0.0143	1	0.0143	5.18	0.0571
	**Interaction Effects**					
	*S*_1_ *S*_2_	0.0008	1	0.0008	0.2840	0.6106
	*S*_1_ *S*_3_	0.0003	1	0.0003	0.0927	0.7696
	*S*_2_ *S*_3_	9.000 × 10^−6^	1	9.000 × 10^−6^	0.0033	0.9561

^a^ Sum of squares; ^b^ degree of freedom; ^c^ mean sum of squares; ^d^ *p*-values < 0.05 were considered to be significant; ns: insignificant.

**Table 5:** Observed and predicted levels for optimal extraction conditions.

Factor	Predicted Optimal Level	Modified Level
*S*_1_ (°C)	63.8	64.2
*S*_2_ (min)	51.6	52.1
*S*_3_ (mL/g)	25.6	25.2
Response	Predicted (%*w*/*w*)	Experimental (%*w*/*w*, *n* = 3)
Sennoside A (*R*_1_)	2.152	2.237 ± 0.051
Sennoside B (*R*_2_)	12.031	12.792 ± 0.475
Aloe-emodin (*R*_3_)	2.331	2.457 ± 0.053
Emodin (*R*_4_)	0.214	0.261 ± 0.0052
Chrysophanol (*R*_5_)	1.411	1.529 ± 0.041

**Table 6:** ABTS and DPPH free radical scavenging activities of SAME samples.

Sample	IC_50_ ABTS (µg/mL)	IC_50_ DPPH (µg/mL)
S1	149.7 ± 5.35	178.93 ± 5.70
S2	301.4 ± 9.76	313.1 ± 9.83
S3	324.7 ± 11.59	343.8 ± 11.27
S4	307.2 ± 11.55	326.7 ± 10.74
S5	58.9 ± 2.33	68.4 ± 2.18
S6	167.5 ± 7.02	192.2 ± 8.39
S7	153.2 ± 6.69	183.8 ± 8.58
S8	60.2 ± 2.19	69.1 ± 2.48
S9	63.1 ± 2.07	74.7 ± 3.44
S10	249.9 ± 10.72	260.1 ± 9.81
S11	197.2 ± 7.53	210.3 ± 7.69
S12	275.2 ± 11.31	287.9 ± 11.77
S13	100.8 ± 3.70	115.4 ± 3.08
S14	61.8 ± 1.83	72.3 ± 3.37
S15	66.7 ± 2.15	76.3 ± 1.89
S16	75.2 ± 2.39	84.8 ± 2.46
S17	306.7 ± 12.05	321.9 ± 12.42
BBD optimized SAME	47.2 ± 1.40	59.7 ± 1.93
Ascorbic Acid	5.9 ± 1.3	6.8 ± 0.9

**Table 7:** Extraction variables selected for BBD optimization.

Independent Variable	Factor Level			Dependent Variable					Goal
	−1	0	+1	Sennoside A yield (% *w*/*w*) (*R*_1_)	Sennoside B yield (% *w*/*w*) (*R*_2_)	Aloe-emodin yield (% *w*/*w*) (*R*_3_)	Emodin yield (% *w*/*w*) (*R*_4_)	Chrysophenol yield (% *w*/*w*) (*R*_5_)	Maximized
Extraction temperature (°C) (*S*_1_)	50	60	70						
Extraction time (min) (*S*_2_)	30	45	60						
Liquid to solid ratio (ml/g) (*S*_3_)	16	24	32						

## Data Availability

Not applicable.
